# Time‐Dependent Recovery of Gastric Emptying After Gastrectomy: A 12‐Month Longitudinal Study Using a ^13^C‐Acetate Breath Test With a Semi‐Solid Meal

**DOI:** 10.1002/ags3.70106

**Published:** 2025-10-06

**Authors:** Sachiko Kaida, Katsushi Takebayashi, Mika Kurihara, Reiko Otake, Haruki Mori, Hiromitsu Maehira, Toru Miyake, Shigeki Bamba, Masaji Tani

**Affiliations:** ^1^ Department of Surgery Shiga University of Medical Science Otsu Shiga Japan; ^2^ Department of Nutrition Care Shiga University of Medical Science Otsu Shiga Japan; ^3^ Department of Fundamental Nursing Shiga University of Medical Science Otsu Shiga Japan

**Keywords:** ^13^C‐acetate breath test, gastrectomy, gastric emptying, long‐term follow‐up, reconstruction method

## Abstract

**Aims:**

Gastric emptying dysfunction is a major concern after gastrectomy for gastric cancer; however, its long‐term course and relationship with surgical procedures remain unclear. This study aimed to evaluate gastric emptying function longitudinally over 12 months postoperatively using the ^13^C‐acetate breath test with a semi‐solid meal.

**Methods:**

A prospective cohort of 95 patients who underwent curative gastrectomy for gastric cancer between April 2021 and July 2024 was assessed. A novel semi‐solid test meal, integrated with a ^13^C‐acetate breath test, was used to evaluate patients' gastric emptying function. Gastric emptying half‐time (T1/2) was measured preoperatively and at 1, 6, and 12 months postoperatively. Patients were stratified according to surgical procedure and reconstruction method.

**Results:**

The median T1/2 was 42.0 min preoperatively, delayed to 45.9 min at 1 month, and then significantly decreased to 23.4 min at 6 months and 18.7 min at 12 months postoperatively. In distal gastrectomy with Billroth I or Roux‐en‐Y reconstruction, T1/2 was prolonged at 1 month but improved markedly at 6 and 12 months. In contrast, proximal gastrectomy with modified side‐overlap esophagogastrostomy and total gastrectomy showed consistently rapid gastric emptying at all time points.

**Conclusion:**

Gastric emptying function after gastrectomy demonstrated dynamic, time‐dependent changes, with an initial postoperative delay followed by significant acceleration within the first year. The pattern and degree of change differ depending on the surgical procedure and reconstruction method. These findings highlight the importance of individualized, and time‐adapted nutritional management after surgery in patients with gastric cancer.

## Graphical abstract

This study examined time‐dependent changes in gastric emptying after gastrectomy for gastric cancer using a ^13^C‐acetate breath test with a semi‐solid meal in 95 patients, revealing that gastric emptying was initially delayed at 1 month but significantly accelerated by 12 months postoperatively. The patterns and degree of change differed by surgical procedure and reconstruction method, highlighting the importance of individualized, time‐adapted nutritional management after gastrectomy.

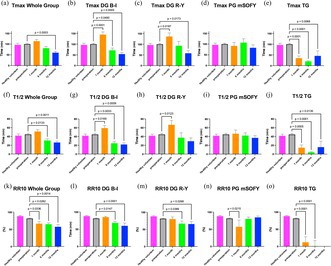

## 1 Introduction

Gastric cancer remains a major cause of cancer‐related mortality worldwide [1], and surgical resection remains the cornerstone of curative treatment. However, the choice of surgical technique and reconstruction method profoundly influences postoperative gastric function, which in turn affects both nutritional status and quality of life [2, 3]. Among postoperative complications, gastric dysfunctions—particularly delayed gastric emptying—poses a significant clinical challenge. Despite its clinical relevance, the mechanisms and temporal progression of postoperative gastric motility remain poorly understood [4, 5].

Previous studies have predominantly evaluated gastric emptying using liquid test meals [6]. Although informative, such methods may not adequately reflect real‐world eating conditions, especially in postgastrectomy patients. Semi‐solid test meals may offer a more physiologically relevant assessment; however, they remain insufficiently studied. Moreover, comparative data on gastric emptying across different types of gastrectomy and reconstruction—such as Billroth I (B‐I), Billroth II, and Roux‐en‐Y (R‐Y) —remain limited despite their critical role in shaping postoperative gastrointestinal physiology.

Recent advances, including the use of the ^13^C‐acetate breath test with semi‐solid meals, have enabled noninvasive and quantitative evaluation of gastric emptying over time [7, 8]. Pilot data from our institution suggested that gastric emptying may be delayed in the early postoperative phase and subsequently accelerated over time, depending on the surgical technique. Although these internal findings were unpublished, they supported the rationale for conducting the present prospective longitudinal study. Notably, patients who undergo distal gastrectomy with B‐I or R‐Y reconstruction typically exhibit accelerated gastric emptying by 6 to 12 months postoperatively. In contrast, patients who undergo proximal or total gastrectomy tend to exhibit consistently rapid gastric emptying, which predisposes them to dumping syndrome.

Several validation studies have demonstrated that the ^13^C‐acetate breath test can reliably assess gastric emptying for liquid and semi‐solid test meals, with significant methodological and physiological considerations depending on the meal consistency [9, 10]. Compared to solid meals, which are influenced by individual differences in mastication, dentition, and salivary flow, semi‐solid test meals can be ingested without chewing and provide a standardized intragastric bolus, thereby enhancing measurement reproducibility. This methodological strength reduces confounding and enhances the reliability of gastric emptying assessment. Despite these advances, there remains a lack of large‐scale, prospective studies evaluating semi‐solid meals with the ^13^C‐acetate breath test in postgastrectomy cohorts across different reconstruction methods and over extended period. According to international dietary guidelines, postgastrectomy patients typically transition from liquid or easily digestible foods to soft or semi‐solid foods as tolerated. Therefore, employing a semi‐solid test meal in this study reflects one stage of common nutritional management after gastrectomy and may enhance the translational relevance of our findings in the context of real‐world clinical practice.

These findings underscore the importance of understanding the dynamic, time‐dependent nature of postoperative gastric motility. Tailoring perioperative nutritional strategies requires insight into both the extent and timing of gastric dysfunction. Therefore, this study aimed to characterize longitudinal changes in gastric emptying over a 12‐months postoperative period, using a ^13^C‐acetate breath test with a novel semi‐solid meal. This study seeks to inform individualized nutritional management and improve long‐term outcomes for patients with gastric cancer by elucidating the influence of surgical and reconstructive approaches on postoperative gastric function.

## 2 Methods

### 2.1 Study Design and Population

This prospective cohort study enrolled 140 patients who underwent curative gastrectomy for histologically confirmed gastric or esophagogastric junction adenocarcinoma at our institution between April 2021 and July 2024 [9, 10]. Eligibility criteria included age ≥ 18 years, Eastern Cooperative Oncology Group (ECOG) [11] performance status ≤ 2, and the ability to undergo serial ^13^C‐acetate breath tests. Exclusion criteria were stage IV disease or recurrence within 12 months, anastomotic obstruction, inability to ingest the test meal, or incomplete follow‐up data. Among the 140 initially enrolled patients, 45 were excluded (loss to follow‐up, *n* = 35; breath test intolerance, *n* = 8; anastomotic obstruction, *n* = 3), resulting in a final analytic cohort of 95 patients. An additional 20 healthy individuals were recruited as controls.

### 2.2 Surgical Procedures and Reconstruction

**FIGURE 1:**
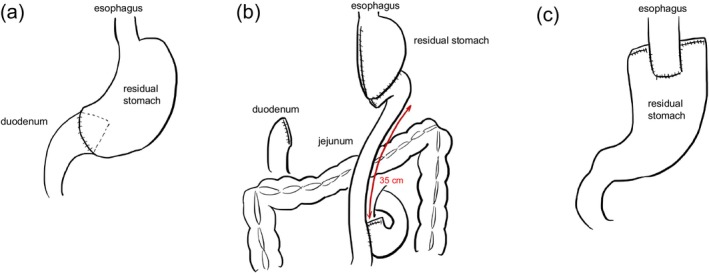
Reconstruction methods following (a) distal gastrectomy, Billroth I, (b) Roux‐en‐Y, and (c) proximal gastrectomy, modified side overlap esophagogastrostomy.

Surgical procedures were performed according to the Japanese Gastric Cancer Association guidelines (3rd edition) [12]. Although the 5th edition of the Japanese Gastric Cancer Association guidelines became available during the study period, our institutional protocols and surgical standardization were based on the 3rd edition at the time of study initiation. Importantly, key principles regarding resection extent and reconstruction methods did not substantially change between the 3rd and later editions, ensuring comparability. Distal gastrectomy (DG) was followed by Billroth I (B‐I) (Figure 1a) or Roux‐en‐Y (R‐Y) reconstruction (Figure 1b), depending on the residual stomach and duodenal anatomy [13, 14]. Proximal gastrectomy (PG) was primarily performed using modified side‐overlap esophagogastrostomy with fundoplication (mSOFY) [15] (Figure 1c), with a minority undergoing double‐tract reconstruction. In the PG cohort, one patient underwent double‐tract reconstruction. Considering its distinct anatomical and physiological characteristics compared to those of mSOFY, this case was excluded from the subgroup analyses but included in the overall PG cohort data only for descriptive statistics. All total gastrectomy (TG) cases were reconstructed using R‐Y. D2 lymphadenectomy was performed for clinical stage II–III or N1–N3 disease, whereas D1+ dissection was applied for clinical stage I cases.

### 2.3 ^13^C‐Acetate Breath Test Protocol

**FIGURE 2:**
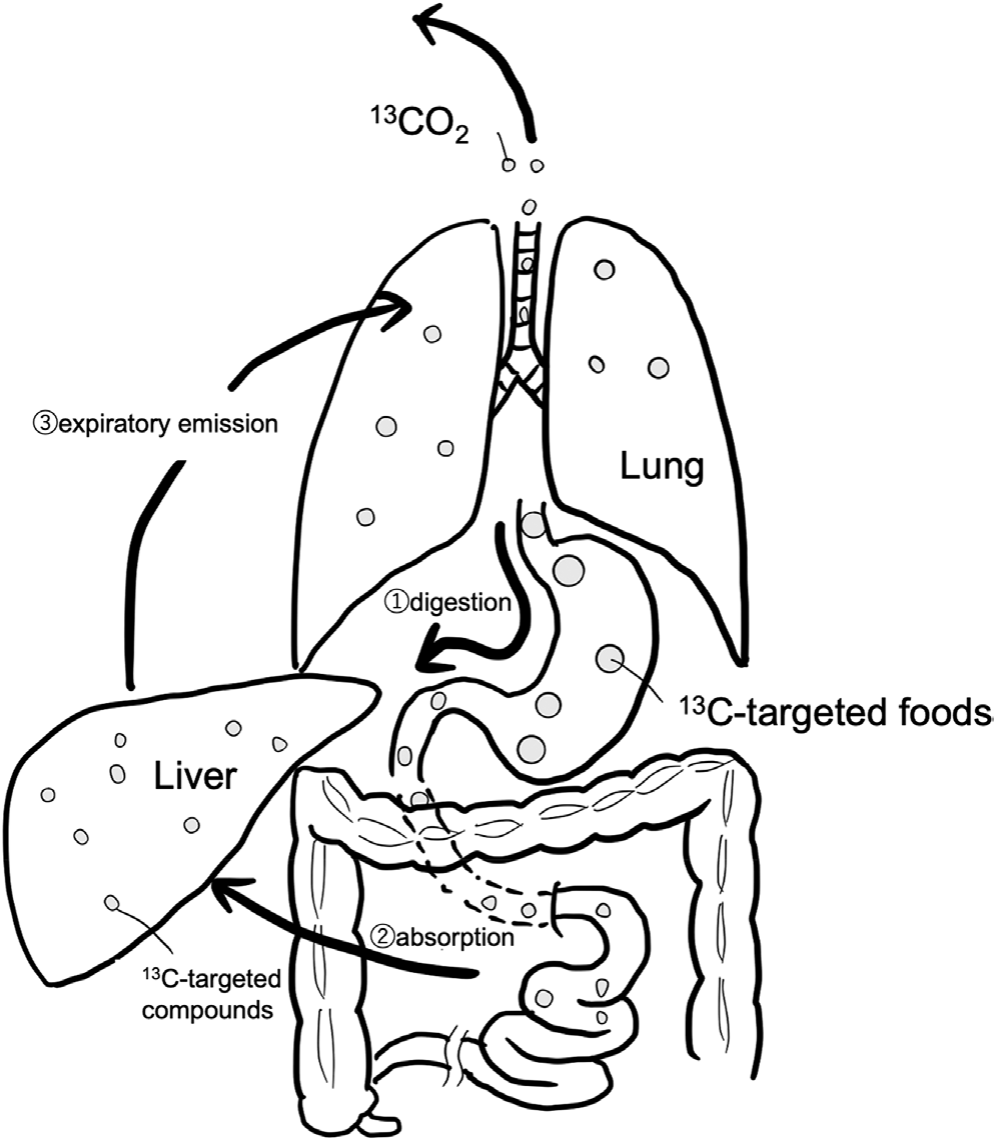
Mechanisms by which food labeled with ^13^C is expelled as exhaled air.

^13^C is a nonradioactive stable isotope of carbon (^12^C). When a ^13^C‐labeled compound (solid or liquid food) is ingested, it passes through the stomach, is absorbed by the small intestine, and is subsequently metabolized by the liver. Since the ^13^C‐labeled compound is rapidly absorbed exclusively through the small intestine, the metabolic rate of the target compound can be measured by monitoring the increase in the ^13^CO_2_/^12^CO_2_ ratio in exhaled breath (Figure 2). A novel semi‐solid test meal was developed consisting of a 200 kcal/100 mL nutritional formula (Isocal, Nestlé Japan Ltd., Tokyo, Japan), 3 g of indigestible dextrin (Clinico, Tokyo, Japan), and 100 mg of ^13^C‐acetate [8, 16]. The semi‐solid meal composition was selected based on previous validation studies demonstrating reproducibility and tolerance in clinical populations [8, 14]. The addition of indigestible dextrin increased viscosity and standardized caloric load, making it suitable for postgastrectomy patients. A test meal was administered to both the patient and control groups. The viscosity of the semi‐solid test meal was measured using a TVB‐10 viscometer (Toki Sangyo, Tokyo, Japan) at 5°C–10°C to ensure consistency across administrations. The test was performed at four time points: preoperatively and at 1, 6, and 12 months postoperatively. Breath samples were collected at baseline and 10, 20, 30, 40, 50, 60, 90, 120, 180, and 240 min after ingestion. Sampling was conducted for up to 240 min, following international recommendations for ^13^C‐acetate breath testing, to accurately capture late‐phase gastric emptying, particularly in distal gastrectomy with R‐Y reconstruction. The ratio of ^13^CO_2_/^12^CO_2_ in exhaled breath was analyzed using an infrared spectrophotometer, and the gastric emptying half‐time (T1/2) was calculated using the Wagner−Nelson analysis method [17, 18] which has been validated against scintigraphy in liquid and semi‐solid meal contexts. Although alternative models exist, we selected this method given its clinical applicability and comparability with existing gastric emptying studies in Japan. Additional parameters included the time to maximum exhalation (*T*_max_) and 10‐min retention rate (RR10), which serve as complementary markers of gastric motility.

### 2.4 Longitudinal Assessment

Each patient underwent a ^13^C‐acetate breath test at baseline (preoperatively) and again at 1, 6, and 12 months postoperatively to evaluate the time‐dependent dynamic of gastric emptying. This longitudinal design allowed intrapatient comparisons and assessment of recovery patterns based on surgical procedures and reconstruction methods.

### 2.5 Clinical and Nutritional Data Collection

Demographic and perioperative variables, including the surgical technique, reconstruction method, and extent of lymph node dissection, were prospectively recorded. Postoperative complications within 30 days were evaluated using contrast‐enhanced computed tomography and fluoroscopy [19], and were classified according to the Clavien–Dindo system [20], with grade ≥ II defined as severe. Body weight, body mass index (BMI), and nutritional parameters were monitored at each follow‐up point.

### 2.6 Statistical Analysis

Continuous variables were expressed as mean ± standard deviation (SD) for normally distributed data, and as median and interquartile range (IQR) for nonnormally distributed data. The normality of continuous variables was assessed using the Shapiro–Wilk test. Sphericity was tested using Mauchly's test; when the sphericity assumption was violated, Greenhouse–Geisser corrections were applied. The Mann–Whitney *U* test was used for between‐group comparisons of nonparametric data, and repeated‐ measures analysis of variance (ANOVA) (with Bonferroni correction for multiple comparisons) was used for longitudinal analysis. The specific statistical tests used for each analysis are described in the figure legends and tables. Missing data were handled using listwise deletion. Statistical significance was set at *p* < 0.05. All analyses were performed using SPSS version 29 (IBM Corp., Armonk, NY, USA) and GraphPad Prism version 10 (GraphPad Software, La Jolla, CA) [21, 22].

## 3 Results

### 3.1 Viscosity of the Test Meals

The viscosities of the nutritive and semi‐solid test meals before the addition of the gelling agent were 57.7 mPa·s at 60 rpm/1 min (8.1°C) for Isocal and 4660 mPa·s at 60 rpm/1 min (8.4°C) for the semi‐solid meal.

### 3.2 Patient Characteristics

A total of 95 patients (67.8%) were eligible for this study based on the inclusion criteria. The patient cohort had a median age of 72 years (range, 40–85 years), with a male‐to‐female ratio of 68:27 and a median body mass index (BMI) of 22.7 kg/m^2^ (range, 15.2–31.7 kg/m^2^) (Table 1). The surgical approaches included open surgery (*n* = 7), laparoscopic surgery (*n* = 29), and robotic surgery (*n* = 59). The types of gastrectomies performed were TG (*n* = 12), DG (*n* = 58), and PG (*n* = 25). For reconstruction, B‐I (*n* = 36) and R‐Y (*n* = 22) were used after DG, whereas mSOFY (*n* = 24) and double‐tract reconstruction (*n* = 1) were performed after PG due to the size of the remnant stomach (Table 2). All TG cases in this study were reconstructed using the R‐Y method. The lymph node dissection levels were D1+ in 52 patients and D2 in 43 patients.

**TABLE 1:** Baseline demographic and clinical characteristics of included patients, excluded patients, and healthy volunteers.

	Healthy volunteers (*n* = 20)		Included patients (*n* = 95)		Excluded patients (*n* = 45)		*p*
Age, year	45	(40–58)	72	(40–85)	74	(43–81)	0.16
Sex							
Male	5	(25.0%)	68	(71.6%)	31	(68.9%)	0.84
Female	15	(75.0%)	27	(28.4%)	14	(31.1%)	
BMI, kg/m^2^	23.3	(17.7–26.1)	22.7	(15.2–31.7)	22.1	(18.2–29.0)	0.61
NLR	—	—	1.64	(0.82–5.61)	2.4	(0.9–8.6)	0.13
Clinical stage							
I	—	—	52	(54.7%)	20	(44.4%)	0.28
≥ II	—	—	43	(45.3%)	25	(55.6%)	
ASA‐PS							
1,2	—	—	89	(93.6%)	42	(93.3%)	> 0.99
≥ 3	—	—	6	(6.4%)	3	(6.7%)	
Adjuvant chemotherapy, *n* (%)	—	—	44	(46.3%)	22	(48.8%)	0.86
Neoadjuvant chemotherapy, *n* (%)	—	—	0	(0%)	0	(0%)	> 0.99
ECOG PS 0–1, *n* (%)	—	—	91	(96%)	41	(91%)	0.27
Diabetes mellitus, *n* (%)	—	—	13	(13.7%)	8	(17.8%)	0.61
Laparoscopic surgery, *n* (%)	—	—	22	(23.1%)	11	(24.4%)	> 0.99
Open surgery, *n* (%)	—	—	5	(5.3%)	2	(4.4%)	> 0.99
Robotic surgery, *n* (%)	—	—	68	(71.6%)	32	(71.1%)	> 0.99
Adjuvant chemotherapy, *n* (%)	—	—	2	(2.1%)	1	(2.2%)	> 0.99

*Note:* Data are presented as median (range) for continuous variables and as number (percentage) for categorical variables. Statistical comparisons were performed using the Mann–Whitney *U* test or Fisher's exact test, as appropriate.

Abbreviations: ASA‐PS: American Society of Anesthesiologists physical status classification; BMI, body mass index; ECOG PS: Eastern Cooperative Oncology Group performance status; NLR, neutrophil‐to‐lymphocyte ratio.

**TABLE 2:** Perioperative and surgical details of the study cohort.

	Patients group (*n* = 95)	
Approaches	Open	7 (7.3%)
	Robotic	59 (62.1%)
	Laparoscopic	29 (30.6%)
Lymph node dissection	D1+	52 (54.7%)
	D2	43 (45.3%)
Operation time (min)		327 (179–498)
Bleeding (mL)		0 (0–488)
*Postoperative complication*		
Clavien‐Dindo Grade	≧ 2	8 (8.4%)
	≧ 3a	3 (3.2%)
Pathological stage	I	45 (47.4%)
	II	22 (23.2%)
	III	23 (24.2%)
	IV	5 (5.2%)
Hospital stay	(POD^a^)	8 (6–51)
Oral intake start	(POD^a^)	2 (2–2)
Mortality		0 (0%)
Operation method	Total gastrectomy	12 (12.6%)
	Distal gastrectomy	58 (61.1%)
	Proximal gastrectomy	25 (26.3%)
Reconstruction	Distal gastrectomy Billroth I	36 (37.9%)
	Roux‐en‐Y	22 (23.2%)
	Proximal gastrectomy modified SOFY^b^	24 (25.3%)
	Double tract	1 (1.1%)
	Total gastrectomy Roux‐en‐Y	12 (12.5%)

*Note:* Values are shown as number (percentage) for categorical variables, and as median (range) for continuous variables.

^a^ Post operative days.

^b^ Side Overlap with Fundoplication by Yamashita.

### 3.3 Time‐Dependent Changes in ^13^CO_2_ Emission

**FIGURE 3:**
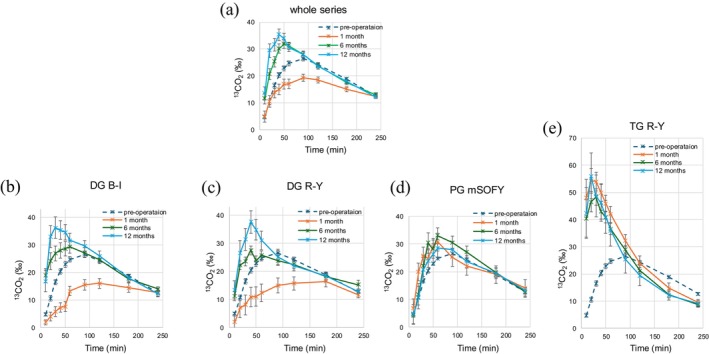
The temporal changes in exhaled ^13^CO_2_ emissions from the intake of semi‐solid test meals to 240 min postbaseline were stratified by surgical method and postoperative time point. In all groups, the dashed line represents preoperative values, orange lines indicate 1 month postoperatively, green lines indicate 6 months postoperatively, and blue lines indicate 12 months postoperatively. (a) Whole series, (b) DG B‐I, (c) DG R‐Y, (d) PG mSOFY, and (e) TG R‐Y group.

Figure 3 presents the temporal profiles of ^13^CO_2_ emissions in expiratory air, measured from baseline to 240 min following ingestion of a semi‐solid test meal, stratified by surgical procedure and postoperative time point. In all groups, the preoperative curve (dashed line) demonstrated a gradual rise in ^13^CO_2_ emissions, peaking between 30 and 90 min after meal ingestion, followed by a subsequent decline.

At 1 month postoperatively, both the DG B‐I and DG R‐Y groups exhibited a delayed and blunted peak in ^13^CO_2_ emissions relative to the preoperative state, indicative of transiently delayed gastric emptying. Notably, at 6 and 12 months after surgery, the emission curves for these groups shifted to earlier and sharper peaks, reflecting a marked acceleration in gastric emptying over time.

In contrast, the PG mSOFY and TG groups consistently demonstrated rapid gastric emptying throughout the postoperative period. The ^13^CO_2_ emission curves in these groups peaked substantially earlier than those observed in the DG groups and remained stable from 1 to 12 months postoperatively, suggesting sustained rapid transit of gastric contents.

Collectively, these findings indicate that the pattern and trajectory of gastric emptying after gastrectomy are strongly influenced by the surgical procedure and reconstruction method. DG with B‐I or R‐Y reconstruction was associated with a transient delay in gastric emptying at 1 month, followed by a pronounced acceleration at later time points. In contrast, PG with mSOFY and TG resulted in persistent rapid gastric emptying beginning in the early postoperative phase.

**FIGURE 4:**
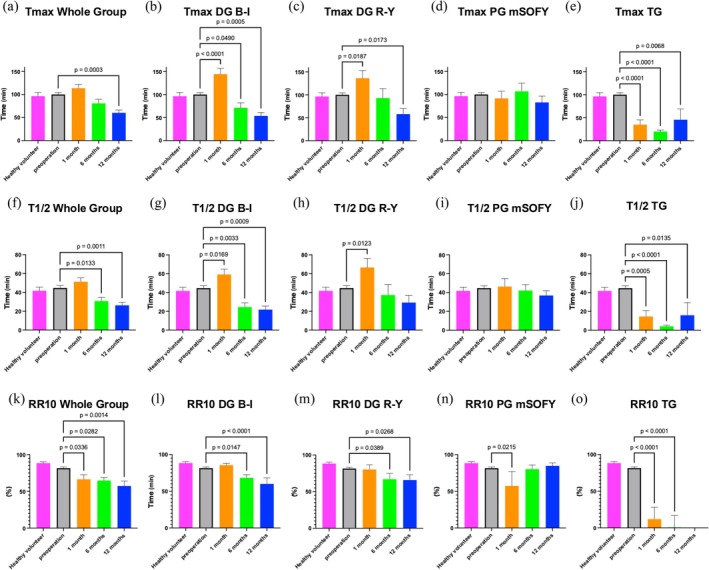
The results comparing temporal changes in key gastric emptying parameters—Time to maximum exhalation (*T*_max_), Gastric Emptying Half‐time (T1/2), and Retention Rate at 10 min (RR10)—between preoperative and postoperative (1, 6, and 12 months) time points for each surgical procedure. (a–e): *T*_max_, (f, j): T1/2 and (k–o): RR10. B‐I, Billroth I; DG, distal gastrectomy; mSOFY, modified side overlap esophagogastrostomy; PG, proximal gastrectomy; R‐Y, Roux‐en‐Y.

Figure 4 presents the results of the Mann–Whitney *U* test comparing temporal changes in key gastric emptying parameters (*T*_max_, T1/2, and RR10) between the preoperative and postoperative (1, 6, and 12 months) time points for each surgical procedure. The upper panels (a–e) show changes in *T*_max_, the middle panels (f–j) show changes in T1/2, and the lower panels (k–o) show changes in RR10. All data are presented as the median (IQR).

### 3.4 Time to Maximum Exhalation (*T*_max_)

For the entire cohort, *T*_max_ did not differ significantly from preoperative values at 1 and 6 months postoperatively but was significantly shorter at 12 months postoperatively (*p* = 0.0003) (Figure 4a). Subgroup analysis revealed that in the DG group, both B‐I and R‐Y reconstructions were associated with a significant prolongation of *T*_max_ at 1 month postoperatively compared to the preoperative values (*p* < 0.0001 and *p* = 0.0187, respectively), followed by a significant shortening at 12 months (*p* = 0.0005 and *p* = 0.0173, respectively) (Figure 4b,c). In contrast, no significant changes in *T*_max_ were observed throughout the study period after PG mSOFY reconstruction (Figure 4d). In the TG group, *T*_max_ was consistently and significantly shorter at 1, 6, and 12 months postoperatively (all *p* < 0.01) (Figure 4e).

### 3.5 Gastric Emptying Half‐Time (T1/2) Over Time

A significant reduction in T1/2 was observed for the entire cohort at 6 and 12 months postoperatively compared to preoperative values (*p* = 0.0133 and *p* = 0.0011, respectively) (Figure 4f). In the DG B‐I group, T1/2 was significantly prolonged at 1 month (*p* = 0.0169) but significantly shortened at 6 and 12 months (*p* = 0.0033 and *p* = 0.0009, respectively) (Figure 4g). Similarly, in the DG R‐Y group, T1/2 was significantly prolonged at 1 month (*p* = 0.0123), but no significant differences were observed at later time points (Figure 4h). No significant changes in T1/2 were observed following PG treatment (Figure 4i). In the TG group, T1/2 was significantly shorter at all postoperative time points (*p* < 0.0005, *p* < 0.0001, and *p* = 0.0135, respectively) (Figure 4j).

### 3.6 Retention Rate at 10 Min (RR10)

RR10 values were significantly lower than the postoperative values at all postoperative time points in the entire cohort (*p* = 0.0336, *p* = 0.0282, and *p* = 0.0014, respectively), indicating a progressive decrease in residual gastric contents 10 min after meal ingestion (Figure 4k). In the DG group, both the B‐I and R‐Y reconstructions showed no significant changes in RR10 at 1 month, however, significant reductions were observed at 6 and 12 months (B‐I: *p* = 0.0147, *p* < 0.0001; R‐Y: *p* = 0.0389, *p* = 0.0268) (Figure 4l,m). In the PG group, a significant decrease in RR10 was observed only at 1 month postoperatively (*p* = 0.0215), with no significant changes at 6 and 12 months (Figure 4n). After TG, RR10 remained extremely low at all time points owing to the absence of the stomach (*p* < 0.0001), further supporting the validity of the assessment (Figure 4o).

## 4 Discussion

This study provides novel insights into time‐dependent changes in gastric emptying function up to 12 months after gastrectomy for gastric cancer, using a semi‐solid test meal combined with the ^13^C‐acetate breath test. While previous reports have focused primarily on early postoperative periods after gastrectomy [23] or pylorus‐preserving pancreatico‐duodenectomy [24], our extended follow‐up revealed a transient delay in gastric emptying at 1 month postoperatively, followed by significant acceleration at 6 and 12 months, particularly in patients undergoing DG B‐I or R‐Y. This dynamic pattern highlights the evolving nature of postoperative gastric motility and suggests that gastric emptying is not static, but adapts over time, aligning with recent long‐term studies on postgastrectomy physiology. Accelerated gastric emptying may predispose patients to dumping syndrome and nutritional deficiencies, whereas transient delays could contribute to early satiety. Although we did not collect direct clinical outcome data in this study, our results suggest possible links between physiologic and clinical consequences, which should be investigated in future studies.

The observed acceleration of gastric emptying at later postoperative stages may result from physiological and anatomical changes such as remodeling of the gastric remnant, alterations in gastric reservoir capacity, and changes in neural and hormonal regulation [25]. These changes can lead to rapid transit of gastric contents into the small intestine, which may predispose patients to dumping syndrome or malabsorption if not properly managed [26]. In contrast, patients who underwent PG mSOFY or TG exhibited consistently rapid gastric emptying throughout the postoperative period, reflecting a smaller or absent gastric reservoir and altered gastrointestinal continuity. These differences underscore the significant impact of surgical techniques and reconstruction methods on postoperative gastric physiology. Postgastrectomy syndrome is believed to result from decreased gastric retention and increased evacuation of the residual stomach; however, these mechanisms are difficult to assess. Traditional methods, such as fluoroscopy and scintigraphy, are invasive and associated with radiation exposure [27, 28]. In contrast, the ^13^C‐acetate breath test offers a noninvasive and simple alternative, validated in multicenter studies [29]. Furthermore, while most prior studies used liquid test meals [17, 18], they may not accurately reflect real‐world food consumption because liquids pass through the stomach more easily by gravity, leading to an overestimation of gastric emptying capacity. In this study, we addressed this limitation by using a semi‐solid test meal that more closely mimics the texture and composition of actual meals. As far as we know, this is the first study to use a semi‐solid meal in conjunction with the ^13^C‐acetate breath test to evaluate long‐term gastric emptying after gastrectomy for gastric cancer.

While previous work confirmed the reliability and validity of ^13^C‐acetate breath testing for liquid and semi‐solid or solid meals, and outlined key differences in emptying kinetics between meal types, most published studies have focused either on nonsurgical or short‐term postoperative populations, or lacked standardized, clinically relevant semi‐solid protocols. Moreover, studies employing solid meals have been limited by high inter‐patient variability secondary to differences in chewing efficiency, dental health, and oral processing function. By using a semi‐solid test meal, our study ensured that all participants received a test meal of uniform viscosity and consistency, thus minimizing methodological confounding and enhancing the rigor of longitudinal assessment across various surgical reconstruction types. Understanding the dynamic, time‐dependent nature of postoperative gastric motility is crucial to tailoring perioperative nutritional strategies. Consequently, this study aims to characterize longitudinal changes in gastric emptying over 12 months after gastrectomy, using a novel semi‐solid meal and the ^13^C‐acetate breath test.

Our findings will inform individualized nutritional management and could improve long‐term outcomes for patients with gastric cancer by clarifying the influence of surgical and reconstructive approaches on postoperative gastric function.

The variation in T1/2 among the different reconstruction methods underscores the significant impact of surgical techniques on postoperative gastric function. Notably, the PG mSOFY group demonstrated a rapid T1/2, suggesting that this reconstruction method allows for quicker passage of ingested food into the small intestine, whereas the DG R‐Y showed the most significant delays, indicating a substantial postoperative functional alteration.

Clinically, these findings emphasize the importance of individualized nutritional guidance and monitoring tailored not only to the type of surgery but also to the postoperative period. Early postoperative care should focus on managing delayed gastric emptying and associated symptoms, whereas later phases should aim to accelerate gastric emptying and address its nutritional consequences. Nutritional interventions may include modifiing of meal consistency, frequency, and composition to mitigate symptoms and optimize nutrient absorption [25, 30]. Despite these valuable insights, this study has several limitations. First, this study was conducted at a single institution, which may have limited the generalizability of the findings. The analytic sample comprised 95 patients, with 45 patients excluded due to loss to follow‐up, breath test intolerance, or anastomotic obstruction, resulting in a 32% attrition rate. Although baseline demographics did not differ significantly between included and excluded patients, selection bias cannot be ruled out; patients lost to follow‐up may have experienced different clinical courses or lower tolerance to repeated testing. Second, several potential confounders, including surgical approach, adjuvant chemotherapy, baseline body mass index, and diabetes mellitus, may influence gastric emptying. Although these factors were recorded in patient demographics, our study was not powered to conduct multivariable adjustment. Therefore, residual confounding cannot be ruled out and should be addressed in larger multicenter cohorts. Third, this extended protocol increased patient burden and may have contributed to the 32% attrition rate. Nonetheless, we judged that 240 min of observation was necessary to accurately evaluate prolonged emptying patterns, particularly in distal gastrectomy with R‐Y reconstruction. However, its feasibility in broader clinical practice, however, may require further evaluation. Fourth, the semi‐solid test meal and the Wagner–Nelson analytical method were chosen based on prior validation studies and their widespread use in Japan. While no absolute gold standard exists for semi‐solid meals in postgastrectomy patients, the Wagner–Nelson method provides an established, reproducible approach that allows comparison with previous literature.

Fifth, our study focused exclusively on physiologic assessment without direct evaluation of clinical symptoms, quality of life, or dumping syndrome severity. Therefore, while physiologic changes may suggest potential clinical consequences, further studies integrating patient‐reported outcomes are needed. While our observations suggest important clinical consequences of altered gastric emptying, definitive links to real‐world outcomes cannot be established from the present data alone. Future well‐powered studies integrating physiologic and outcome measures are warranted. Finally, the observational study design limited our ability to establish causal relationships between the type of reconstruction and observed outcomes. Randomized controlled trials are needed to confirm causality and eliminate potential confounding factors.

## 5 Conclusion

These findings suggest that gastric emptying changes dynamically in a time‐dependent manner after gastrectomy. Our results provide a foundation for future development of perioperative nutritional strategies tailored to these changes. However, future studies integrating physiologic measurements with clinical outcomes will be necessary to determine how such dynamics may inform individualized, time‐adapted nutritional management.

## Author Contributions

**Sachiko Kaida:** project administration, writing – review and editing, writing – original draft, resources, data curation, software, validation, investigation, conceptualization, methodology, formal analysis, visualization, funding acquisition. **Katsushi Takebayashi:** data curation, conceptualization, validation. **Mika Kurihara:** data curation, visualization, methodology, conceptualization. **Reiko Otake:** data curation, supervision. **Haruki Mori:** data curation, supervision. **Hiromitsu Maehira:** data curation, supervision. **Toru Miyake:** data curation, validation, supervision. **Shigeki Bamba:** supervision, writing – review and editing. **Masaji Tani:** supervision, writing – review and editing.

## Ethics Statement

The protocol for this research project was approved by a suitably constituted Ethics Committee of the institution and it conformed to the provisions of the Declaration of Helsinki. The Research Ethics Committee of the Shiga University of Medical Science approved this study (approval no. R2020‐149). All informed consent was obtained from the research volunteers.

## Conflicts of Interest

The authors declare no conflicts of interest.
